# Deductively coding psychosocial autopsy interview data using a few-shot learning large language model

**DOI:** 10.3389/fpubh.2025.1512537

**Published:** 2025-02-19

**Authors:** Elias Balt, Salim Salmi, Sandjai Bhulai, Stefan Vrinzen, Merijn Eikelenboom, Renske Gilissen, Daan Creemers, Arne Popma, Saskia Mérelle

**Affiliations:** ^1^Research Department, 113 Suicide Prevention, Amsterdam, Netherlands; ^2^Department of Psychiatry, Amsterdam University Medical Centers, Amsterdam, Netherlands; ^3^Department of Mathematics, VU University, Amsterdam, Netherlands; ^4^Department of Clinical Psychology, Leiden University, Leiden, Netherlands; ^5^Department of Child- and Youth Psychiatry, GGz Oost-Brabant, Boekel, Netherlands; ^6^Department of Psychiatry and Psychosocial Care, Radboud University Nijmegen, Nijmegen, Netherlands; ^7^Department of Child and Adolescent Psychiatry and Psychosocial Care, Amsterdam University Medical Center, Amsterdam, Netherlands

**Keywords:** qualitative research, psychosocial autopsy, large language model (LLM), suicide prevention, public health

## Abstract

**Background:**

Psychosocial autopsy is a retrospective study of suicide, aimed to identify emerging themes and psychosocial risk factors. It typically relies heavily on qualitative data from interviews or medical documentation. However, qualitative research has often been scrutinized for being prone to bias and is notoriously time- and cost-intensive. Therefore, the current study aimed to investigate if a Large Language Model (LLM) can be feasibly integrated with qualitative research procedures, by evaluating the performance of the model in deductively coding and coherently summarizing interview data obtained in a psychosocial autopsy.

**Methods:**

Data from 38 semi-structured interviews conducted with individuals bereaved by the suicide of a loved one was deductively coded by qualitative researchers and a server-installed LLAMA3 large language model. The model performance was evaluated in three tasks: (1) binary classification of coded segments, (2) independent classification using a sliding window approach, and (3) summarization of coded data. Intercoder agreement scores were calculated using Cohen’s Kappa, and the LLM’s summaries were qualitatively assessed using the Constant Comparative Method.

**Results:**

The results showed that the LLM achieved substantial agreement with the researchers for the binary classification (accuracy: 0.84) and the sliding window task (accuracy: 0.67). The performance had large variability across codes. LLM summaries were typically rich enough for subsequent analysis by the researcher, with around 80% of the summaries being rated independently by two researchers as ‘adequate’ or ‘good.’ Emerging themes in the qualitative assessment of the summaries included unsolicited elaboration and hallucination.

**Conclusion:**

State-of-the-art LLMs show great potential to support researchers in deductively coding complex interview data, which would alleviate the investment of time and resources. Integrating models with qualitative research procedures can facilitate near real-time monitoring. Based on the findings, we recommend a collaborative model, whereby the LLM’s deductive coding is complemented by review, inductive coding and further interpretation by a researcher. Future research may aim to replicate the findings in different contexts and evaluate models with a larger context size.

## Background

Qualitative research is essential for suicide prevention to establish new theories, explore trends and identify previously unexplored psychosocial risk factors (1). Aspers and Corte (2) broadly define qualitative research as: *“an iterative process in which improved understanding to the scientific community is achieved by making new significant distinctions resulting from getting closer to the phenomenon studied.”* More specifically, Tenny and colleagues (3) note that, instead of collecting numeric data points, qualitative research aims to gather participants’ perceptions, experiences or behavior. Notwithstanding its importance, qualitative research has received criticism for being prone to interpretation bias, problems with accuracy, and challenges to reproducibility (4). Moreover, there are various labor-intensive tasks involved with qualitative research, particularly when interviews are involved. These range from generating full-word transcriptions to coding the data, which entails that the text is labeled along certain concepts to provide structure and facilitate subsequent analysis (1). While quantitative analyses have been largely automated (e.g., statistical tests), many processes in qualitative research remain tediously archaic.

Automating part of qualitative analyses by means of artificial intelligence may reduce interpretation bias, enhance the reproducibility of findings from qualitative research, and reduce the researchers’ time investment. In 2011, Yu and colleagues (5) already noted that text processing tools can “*capitalize on the epistemological compatibility between text mining and qualitative research,”* and this is echoed by a recent editorial (6). Over the last years, large language models (LLM) have been recognized for their ability to generate general-purpose language. LLMs are transformer models (7) that were trained with an exceptionally large corpus of linguistic data and can interpret text, respond to queries, and generate textual content in a human-like fashion within the reasonable limitations of the training data (8). Several benchmarks for zero-shot questions (meaning the LLM had no prior knowledge of the question) show that LLMs can perform at high levels of accuracy. LLMs may therefore have the potential to analyze large amounts of textual data in- and outside of scientific research, for example in sentiment analysis (9), summarization of content, e.g., medical records in clinical settings (10), and structuring textual data (11).

Several recent studies concluded that integrating LLMs with qualitative analysis could have merit to make qualitative research more efficient and replicable, particularly in the coding process (11–13). In a recent study, Xiao and colleagues (11) used a pretrained Chat-GPT based language model to deductively code qualitative data from interviews with children. Their model achieved fair to substantial agreement with the coding work of an independent researcher. There are other examples of successfully integrating an LLM in qualitative analyses (12, 14). However, Christou and colleagues (15) provide important critical reflections on the pitfalls of using language models to support qualitative analysis, including hallucination, which entails that the model provides output that is incorrect or nonexistent, as well as bias (models can consistently fail to address a particular theme due to deficient training and performance in that topic). Consequently, the authors suggest that an LLM cannot perform independently of researchers yet. Moreover, experts of qualitative research warn that LLMs should be used cautiously and that ethical and intellectually challenging tasks are still beyond their potential (16). This conceivably includes inductive coding, whereby the model must recognize, prioritize and label themes in a text body without receiving *a priori* definitions. A collaborative approach may, however, be feasible, whereby the model performs deductive coding, which is scrutinized by a research team (11). This could support public health surveillance in novel ways, as noted by Olawade and colleagues in a recent narrative review (17).

Psychosocial autopsy is a research methodology aimed at investigating suicide cases, which generally combines interviews with bereaved informants and available records (18), facilitating mixed methods analyses with a large qualitative component. Internationally, psychosocial autopsy is an acclaimed method to obtain insights into risk factors for suicide, in particular in a case–control design (19, 20). Additionally, psychosocial autopsy interviews can be used to obtain a better understanding of proximal stressors for suicide (21). In the Netherlands, psychosocial autopsy studies have been formerly conducted to investigate suicides of young people (22) and railway suicides (23). Recently, a psychosocial autopsy has been implemented to monitor and investigate suicides routinely on a national scale. This resulted in a cross-sectional, retrospective, dynamic cohort where quantitative and qualitative data are added constantly. This data envelops dichotomous and categorical variables such as psychiatric diagnoses or education level, but also data from many interviews with next-of-kin of suicide decedents. Through this information, we can obtain real-time insights into psychosocial characteristics associated with suicides, investigate trends, and provide recommendations for prevention.

LLMs may be integrated with research procedures to standardize the qualitative analysis of psychosocial autopsy data, to increase efficiency and possibly enhance reproducibility. To the best of our knowledge, state-of-the-art language models have not been evaluated for their ability to process complex interview data from a psychosocial autopsy. We can understand the possibilities of LLMs in this field better based on empirical data in addition to expert authority and theoretical knowledge. The current study aimed to investigate if an LLM achieves sufficient agreement with a researcher in the deductive coding of interview data obtained in a psychosocial autopsy and can summarize data of adequate quality to be feasibly integrated with qualitative research procedures.

## Methods

### Data and procedures

The corpus for this study consisted of 38 full-word interview transcripts obtained in a psychosocial autopsy study of railway suicides, collected between October 2021 and May 2022 (MREC of the Amsterdam University Medical Centre, NL76295.029.21). Respondents were people who lost a loved one to suicide. The instrument for the two-hour semi-structured interviews was based on international examples and covered topics ranging from school- and work problems to social media use and mental health problems, using instruments based on international examples (19, 20, 24) and national health monitors (25). Instead of training a model to recognize codes, we employed a few-shot learning approach (26). This approach included a labeled example of each class in the prompt. This methodology was guided by advancements in the field which suggest that LLMs can recognize topics without specific training due to their extensive knowledge of contextual semantics (8). For our study, we used the 70B instruct version of Llama3. Llama3 is an open-source, large language model which was developed by Meta (27). The model uses generative artificial intelligence for language prediction. Version 3 of the model had high benchmark scores in various language processing tasks and an increased context size compared to earlier versions of the model. Most importantly, the model could be installed onto a server for offline use, which ensured safety of the data, and this was a prerequisite for this study. The model was sourced and implemented using the Huggingface Transformers library (28). We opted for this model over other open-source models as it had the best performance in language understanding at the time of development (April 2024), as is reflected by benchmarks in Table 1.

**Table 1:** List of language understanding benchmarks for Llama 3 70b instruct.

Benchmark		Score
LiveBench	Average	48.9
	Language comprehension	42.4
GPQA		39.5
MMLU		82.0

The topics the model extracted were segments of text associated with predefined labels, or codes, which are commonly used in qualitative analyses. The dataset contained 2,666 coded transcript fragments averaging 207.62 (SD 190.57) words. The transcripts had been coded by researchers using a coding sheet. Manual coding of the interviews was performed in the context of the psychosocial autopsy study and took place from May 2022 to September 2022. Coding was performed independently by two individual researchers and received the consensus of a third researcher. This represented the ‘gold standard.’ Both deductive coding, which means that codes were formulated *a priori*, and inductive coding, which entails that new codes were created for emerging themes, were performed by the researchers. However, for the model’s evaluation we focused on deductive codes, formulated directly from questions in the interview instrument with fixed definitions. The code list with corresponding definitions has been supplemented with this manuscript (Supplementary File 1).

### Prompt engineering

Before the formal evaluation, we ran several preliminary iterations to optimize prompts and prevent logical errors. We first used synthetic interview data, after which we dedicated a single transcript of the corpus to engineer the prompt. Furthermore, one positive and one negative example were selected from the coded transcript fragments for each code. These examples were used to create the few-shot learning prompt and excluded from the evaluation dataset. We started the evaluation once the research team agreed that the prompts were satisfactory.

To implement the few-shot classification approach, the following prompts were utilized:

System Prompt: This prompt defines the intended behavior of the LLM and sets the context for its responses. The system prompt used was:
"You are a helpful assistant. Your task is to determine whether a given topic is explicitly mentioned in segments of interview transcripts between interviewers and relatives of suicide victims. Respond with 'True' or 'False,' initially assuming 'False' unless the text explicitly proves otherwise."
User Prompt: This prompt specifies the classification question. The prompt used was:
"Could you please tell me if the topic '{label_definition}' is mentioned in the interview transcript: '{interview_text}'?"

To create a few-shot learning prompt, correct responses from the LLM were artificially included for the first two examples in the prompt. These examples served as demonstrations of the desired input–output behavior. The LLM was then asked to generate the response for a third, unseen example based on the context provided. The final prompt for the few-shot setting was constructed as follows:

System: “You are a helpful assistant. Your task is to determine whether a given topic is explicitly mentioned.”
User: “Could you please tell me if the topic ‘{label_definition}’ is mentioned in the interview transcript: ‘{positive_interview_text}’?”
Assistant: “True.”
User: “Could you please tell me if the topic ‘{label_definition}’ is mentioned in the interview transcript: ‘{negative_interview_text}’?”
Assistant: “False.”
User: “Could you please tell me if the topic ‘{label_definition}’ is mentioned in the interview transcript: ‘{unseen_interview_text}’?”

### Model evaluation

The evaluation foremostly concerned the model’s capability. Still, the research team also discussed the model’s utility, transparency and accountability of the LLM in the context of psychosocial autopsy research (29).

We evaluated the model’s capability on three levels, moving toward increasingly specialized tasks. Aligning the pioneering work of Xiao and colleagues (11), we aimed to assess the ability of the model to:

1. Correctly identify a code in a segment.
2. Identify segments in the transcript that match a specific code.
3. Summarize relevant information from identified segments with respect to a specific code.

### Level 1: binary classification

In the first level we introduced a binary classification problem. The model was prompted to determine whether a specific code was found in a text segment. The code in this context is the detailed description of a topic. There was agreement if both the researcher and the model determined that a code was (TP), or was not (TN), found in the text. We calculated accuracy and intercoder agreement (Cohens Kappa). Landis and Koch (30) propose criteria for agreement between qualitative researchers, with a Cohens Kappa of less than 0 as indicating no agreement, between 0 and 0.20 as slight, 0.21 and 0.40 as fair, 0.41 and 0.60 as moderate, 0.61 and 0.80 as substantial, and 0.81 and 1 as nearly perfect agreement.

### Level 2: identify segments in a transcript

For the second step, the model was presented with the same prompt, i.e., classify text based on a topic definition, but applied to the entire transcript using a sliding window approach. The windows were subsequent sentences in the transcript that did not exceed 300 words. The starting sentence of the next window was at least 75 words after the starting sentence of the current window. Motivations for this approach were that (1) the corpus was too long to integrally load into the model, (2) the interviews lacked logical markers to segment the text, and (3) there was a high variance in the length of fragments coded by the researchers. During pretests, we confirmed that segment length did not affect model performance, except when segments were either very short or very long. A length of 300 words was deemed appropriate, accounting for model performance and computational limits.

**Figure 1:**
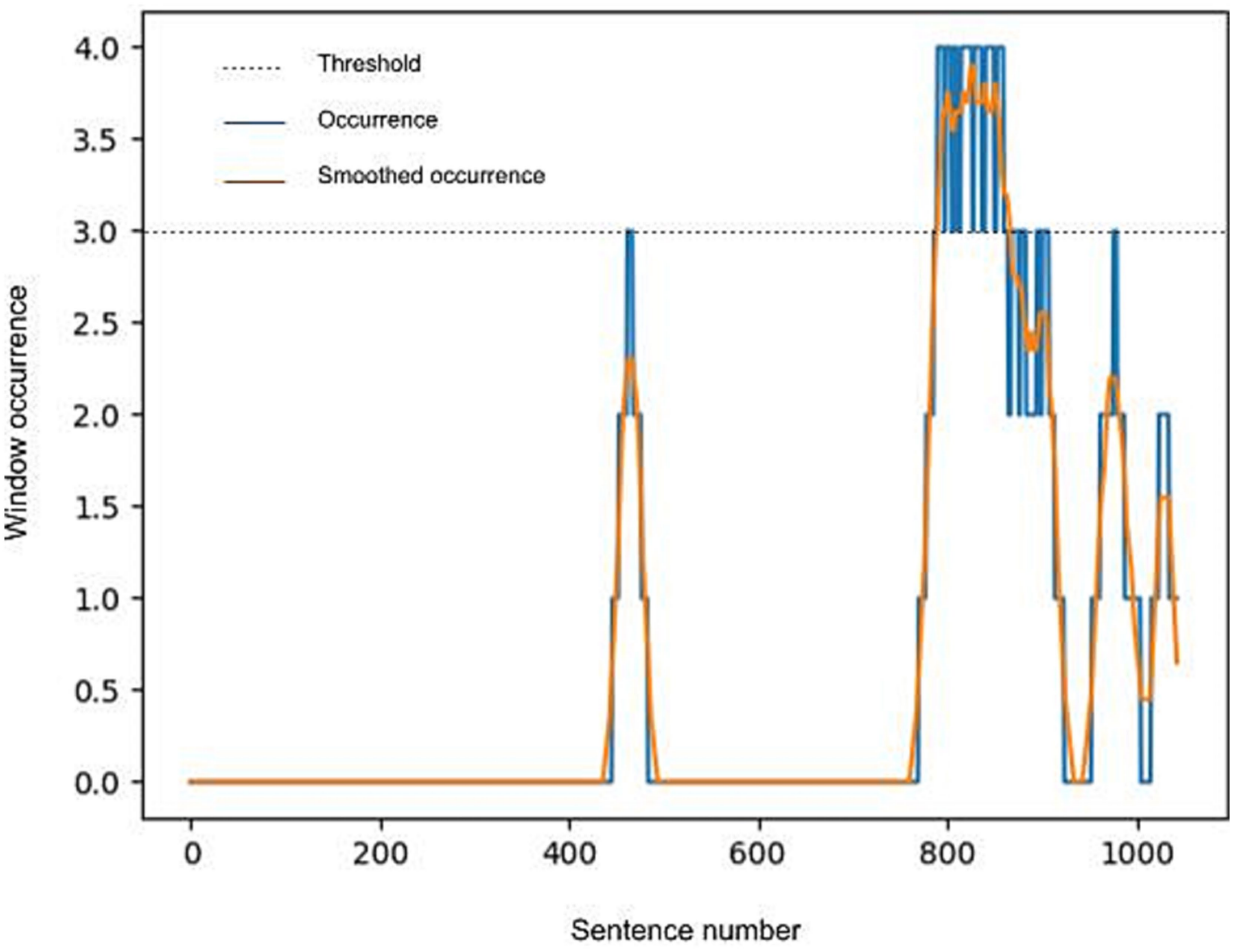
Example peak detection and smoothing procedure.

After the sliding window operation, peak detection was performed to filter any text relevant to a code from the interview. Peak detection was done by taking the number of times a sentence appeared in a positively classified window, smoothing this number using a moving average, and selecting all sentences where the result was above a threshold. The threshold was initially set at 51% of the maximum window overlap, rounded up to the next whole number. In the case of our parameters, this was a maximum of four, thus the threshold was set at three. Selected sentences that formed contiguous blocks of text were grouped. Each group obtained in this way is one detected segment. However, if a particular code resulted in large segments that exceeded the capacity of the model, the threshold was incrementally increased, until the largest segment for that code was 3,000 words at maximum. This was a prerequisite for the summarization task (Figure 1).

We evaluated this task based on two metrics. The relative code frequency of the LLM (i.e., how many segments were detected) was compared to that of the researcher. Because of the sliding window, we did not expect the frequency of codes labeled by the model and the researcher to be similar. Instead, we report the relative frequency. This measure indicates how likely the model was to identify a code compared to the researcher and thus illustrates the model’s tendency to emphasize particular codes. Second, we compared the binary occurrence of codes in the matrices of the researcher and the model and calculated accuracy.

### Level 3: summarization

Finally, we evaluated a summarization task. The model was prompted to summarize all identified segments per code. If too much text was identified for a single prompt, the segments were equally divided into two or more prompts. As part of the qualitative analysis method ‘Constant Comparative Method’ (31), researchers created a matrix with interview cases in rows, and codes in columns, facilitating axial comparison of key themes between cases. Data coded by the researcher was summarized into this matrix. A duplicate matrix was developed with the data coded by the model. Two researchers independently assessed the content of the model’s summaries compared to the researchers’ summaries. The researchers scored the LLM summaries with ‘good,’ ‘adequate,’ or ‘poor.’ Additionally, notable qualitative findings were recorded and discussed with the research team.

### Data analysis

For the binary classification task, we calculated the sensitivity, specificity, accuracy and Cohens Kappa. For the code frequencies obtained using the sliding window method we report the code frequency of the LLM, of the researcher, and a ratio of the code frequency of the LLM compared to that of the researcher (Factor). Finally, we report the classification score of the sliding window in terms of accuracy.

## Results

### Binary classification

The model had a mean accuracy of 0.84 (SD: 0.09) in binary classification. There was substantial agreement with the researcher (Cohens Kappa: 0.68). The highest accuracy scores were found for codes detailing a specific topic, including details about the location and circumstances of the suicide, transportation to the location, the suicide method and preparation of the suicide. We found a lower performance for codes that aimed to capture (changes in) behaviors, communication and interpersonal problems. Specifically, we identified a high number of false positives compared to a low number of false negatives. This means the model labeled data with a code that the researcher did not use. Table 2 reports the model’s performance in the binary classification task, with sensitivity, specificity, accuracy (ACC) and intercoder agreement (Cohens Kappa, CK).

**Table 2:** Binary classification task.

Code name	Count	TP	TN	FP	FN	Sens.	Spec.	ACC	CK
Travel to site_car	24	12	12	0	0	1.00	1.00	1.00	1.00
Sui prep_left significant object	34	17	16	1	0	0.94	1.00	0.97	0.94
Sui prep_substance use	98	47	47	2	2	0.96	0.96	0.96	0.92
Travel to site_bicycle	36	17	17	1	1	0.94	0.94	0.94	0.89
Sui prep_gather information	114	54	53	4	3	0.93	0.95	0.94	0.88
Travel to site_pub. trans.	12	6	5	1	0	0.86	1.00	0.92	0.83
Sui_comm_farewell note	212	106	88	18	0	0.85	1.00	0.92	0.83
Sui_comm_social media	46	23	19	4	0	0.85	1.00	0.91	0.83
Travel to site_other	10	5	4	1	0	0.83	1.00	0.90	0.80
Sui_comm_written	68	33	28	6	1	0.85	0.97	0.90	0.79
Location_characteristics	220	109	88	22	1	0.83	0.99	0.90	0.79
Sui prep_alone at time of death	70	30	32	3	5	0.91	0.86	0.89	0.77
Sui prep_scouting at location	100	41	47	3	9	0.93	0.84	0.88	0.76
Travel to site_walk	48	22	20	4	2	0.85	0.91	0.88	0.75
Location_open tracks	76	32	34	4	6	0.89	0.85	0.87	0.74
Location_proximity	98	49	35	14	0	0.78	1.00	0.86	0.71
Sui prep _practical matters	104	40	47	5	12	0.89	0.80	0.84	0.67
Last_contact	242	121	81	40	0	0.75	1.00	0.84	0.67
Location_familiarity	66	33	22	11	0	0.75	1.00	0.83	0.67
Last_months_appearance	82	27	41	0	14	1.00	0.75	0.83	0.66
Method_motivation	140	67	49	21	3	0.76	0.94	0.83	0.66
Last_months_healthcare	468	224	163	71	10	0.76	0.94	0.83	0.65
Sui prep_planning	76	27	35	3	11	0.90	0.76	0.82	0.63
Location_station	26	13	8	5	0	0.72	1.00	0.81	0.62
Sui_method_circum.	202	93	68	33	8	0.74	0.89	0.80	0.59
Last_months_contact	886	407	278	165	36	0.71	0.89	0.77	0.55
Last_months_ideation	340	157	104	66	13	0.70	0.89	0.77	0.54
Last_months_relation	448	182	160	64	42	0.74	0.79	0.76	0.53
Last_months_behav. change	326	155	89	74	8	0.68	0.92	0.75	0.50
Last_months_mood	708	341	183	171	13	0.67	0.93	0.74	0.48
Last_months_ALE	332	97	142	24	69	0.80	0.67	0.72	0.44
Hist_suicide related comm.	384	160	85	107	32	0.60	0.73	0.64	0.28
Last_months_behaviour	778	365	118	271	24	0.57	0.83	0.62	0.24
Total/mean	6,874	3,112	2,218	1,219	325	0.82	0.91	0.84	0.68

### Sliding window code frequencies

Table 3 shows the relative frequency of codes labeled by the LLM compared to the researchers. The LLM was more likely to determine a code in a corpus by a factor 1.6. Seemingly paradoxical, the absolute code frequency was higher among the researchers. Codes that the LLM was disproportionally inclined to use were often codes with overall low occurrence, leading to a high relative frequency but a small difference in the absolute number of classified segments. These included multiple codes relating to the mode of transportation used by the decedent to travel to the location of the suicide. The median factor was 0.8.

**Table 3:** Sliding window classification (frequencies).

	LLM (code frequency)	Researcher (code frequency)	Factor (LLM/researcher)
Travel to site__other	77	6	12.8
Travel to site__pubtrans	56	7	8.0
Location_station	47	14	3.4
Travel to site__car	29	13	2.2
Location_familiarity	72	34	2.1
Sui comm_social media	45	24	1.9
Sui comm_written	61	35	1.7
Location_proximity	82	50	1.6
Sui prep_left significant object	29	18	1.6
Method_motivation	97	71	1.4
Sui prep_planning	48	39	1.2
Travel to site_bicycle	23	19	1.2
Travel to site_walking	24	25	1.0
Last_contact	115	122	0.9
Sui prep_gather information	47	58	0.8
Sui prep_substance use	40	50	0.8
Last_months_behav. change	128	164	0.8
Location_open tracks	29	39	0.7
Last_months_SI	124	171	0.7
Sui prep_alone at time of death	26	36	0.7
Sui_method_circum.	70	102	0.7
Sui prep_scouting at location	34	51	0.7
Last_months_behaviour	240	390	0.6
Last_months_relation	127	225	0.6
Last_months_healthcare	116	235	0.5
Sui prep_practicalmatters	25	53	0.5
Last_months_appearance	19	42	0.5
Location_characteristics	44	111	0.4
Sui comm_farewellnote	42	107	0.4
Last_months_mood	139	355	0.4
Last_months_contact	127	444	0.3
Last_months_ALE	26	167	0.2
Hist_sui comm	26	193	0.1
Total/mean	2,234	2,663	1.6

### Sliding window classification

We plotted the dichotomous occurrence of codes identified by the LLM with the sliding window approach. This was compared to the work of the researcher (Table 4). The model’s accuracy in this task was 0.67. It performed well for most codes, with several outliers that strongly influenced overall performance, including the mode of transportation to the location of the suicide. Some performance scores contrasted those found in the binary classification, which we address in the discussion.

**Table 4:** Sliding window classification (binary).

	TP	TN	FP	FN	ACC
Last_months_mood	38	0	0	0	1.00
Sui_method_circum.	38	0	0	0	1.00
Last_months_behaviour	37	0	0	1	0.97
Last_months_contact	37	0	0	1	0.97
Last_contact	36	0	2	0	0.95
Method_motivation	36	0	2	0	0.95
Location_characteristics	33	0	0	5	0.87
Last_months_relation	31	1	5	1	0.84
Last_months_ideation	32	0	3	3	0.84
Location_proximity	32	0	6	0	0.84
Last_months_behav. change	30	0	7	1	0.79
Location_open tracks	21	8	3	6	0.76
Travel to site_bicycle	8	21	6	3	0.76
Last_months_healthcare	25	3	4	6	0.74
Travel to site_car	9	18	11	0	0.71
Sui_prep_information	24	3	6	5	0.71
Sui prep_practical matters	8	19	3	8	0.71
Sui comm_farewellnote	21	6	3	8	0.71
Travel to site_walk	12	14	8	4	0.68
Sui prep_left significant object	5	19	14	0	0.63
Last_months_appearance	8	14	3	13	0.58
Sui prep_scouting at location	17	5	8	8	0.58
Location_familiarity	20	1	16	1	0.55
Sui prep_substance use	20	1	3	14	0.55
Sui prep_alone at time of death	12	9	6	11	0.55
Hist_sui comm	16	4	2	16	0.53
Sui prep_planning	7	10	16	5	0.45
Last_months_ALE	17	0	1	20	0.45
Location_station	7	8	23	0	0.39
Sui comm_written	10	5	19	4	0.39
Sui comm_SoMe	9	4	18	7	0.34
Travel to site_pubtrans	5	3	30	0	0.21
Travel to site_other	3	0	35	0	0.08
Total	664	176	263	151	0.67

### Summarization

Table 5 presents the scores for the LLM’s summaries of the data. Eighty percent of the summaries were rated as ‘adequate’ or ‘good.’ and sufficiently rich for subsequent qualitative analyses. It repeatedly referenced the original text, without having been specifically prompted, which facilitated review. Illustrative (anonymized) examples of a good, adequate and poor summary have been added as Supplementary File 2.

**Table 5:** Summarization task rated.

	First rater			Second rater		
Code	Good	Adequate	Poor	Good	Adequate	Poor
Sui comm_farewellnote	34	2	2	22	7	9
Last_months_contact	31	4	3	20	17	1
Last_months_mood	31	6	1	22	13	3
Location_familiarity	31	4	3	28	7	3
Location_open tracks	31	2	5	32	0	6
Sui prep_alone at time of death	30	3	5	23	3	12
Last_months_appearance	29	2	7	26	4	8
Travel to site_bicycle	29	2	7	31	0	7
Location_characteristics	29	5	4	27	6	5
Sui_method_circum.	28	9	1	24	12	2
Sui prep_practical matters	28	1	9	15	12	11
Last_months_relation	27	8	3	18	16	4
Travel to site_car	27	1	10	27	3	8
Last_months_behaviour	26	8	4	24	11	3
Last_months_healthcare	26	5	7	22	6	10
Travel to site_walk	26	1	11	22	7	9
Sui prep_left significant object	26	11	1	29	5	4
Last_contact	25	12	1	20	14	4
Last_months_SI	25	9	4	22	14	2
Location_proximity	25	10	3	30	7	1
Last_months_behav. change	24	9	5	20	14	4
Sui prep_planning	24	7	7	31	4	3
Sui comm_SoMe	24	11	3	26	9	3
Sui prep_scouting at location	20	8	10	24	2	12
Sui prep_substance use	19	14	5	25	8	5
Location_station	18	10	10	19	12	7
Sui prep_information	18	11	9	21	9	8
Sui comm_written	16	19	3	21	13	4
Method_motivation	15	19	4	11	22	5
Travel to site_other	8	19	11	4	23	11
Travel to site_Pubtrans	8	10	20	8	12	18
Hist_sui comm	6	4	28	3	1	34
Last_months_ALEs	0	3	35	2	2	34
Total	764	249	241	699	295	260
Percentage	61%	20%	19%	56%	24%	21%
Mean	23.2	7.5	7.3	21.2	8.9	7.9
SD	8.0	5.1	7.3	7.9	5.9	7.7

The researchers qualitatively analyzed the summaries and marked key themes that constituted adequate and poor summaries.

### Unsolicited elaboration

For all codes, including those with narrow definitions, the LLM was inclined to return summaries that were far more elaborate than the code definition would stipulate. This occurred in at least 247 of the coded segments (19.7%) As opposed to hallucination (theme 4), unsolicited elaboration referred to data that was both correct and relevant output, but not for a given code. This produced duplicate data under multiple codes. Segments typically included information to which various codes could be applied, but the model should remove abundant information in the summary when given a focal point.

### Interpretation

The model did not consistently recognize the subject of a sentence, which compromised the credibility of some details in the summaries. The model may have been confused by different demonstrative pronouns used by the interviewer and the respondent. The fact that transcripts were anonymized during transcription may have also affected this.

### Failure to report

The model failed to summarize crucial information under the correct code in at least 38 instances across the 1,254 fragments (3.0%). For example, in one case, the model did not recognize that a young man had lied about having to go to school on the day he took his own life. In fact, the model mentioned that the boy had to attend school on the day of his suicide, seemingly oblivious to semantic cues referring to this as a lie. However, it stood out that the LLM sometimes reported the required information under an overlapping code. In the case example, the model reported the decedent lying about his school assignment under another code. Overlapping codes (and definitions) appeared to have acted as a failsafe at the cost of accuracy.

### Hallucination

In at least sixteen summarized segments (1.3%), the model fabricated a summary that was factually wrong or based on non-existent data. Notably, it stood out that the hallucinated data was clearly not in line with the quality of the other output, which made it easy to recognize. Nevertheless, this finding indicates a need to consistently check the LLM’s work, which has obvious implications for the feasibility of independent LLM data classification.

### Chronological assessment

The LLM performed poorly at summarizing codes relying on a non-specific time indicator. An example was the code ‘Last_contact’ which refers to the last time the respondent saw or spoke to the decedent. The LLM wrongly summarized an interaction as ‘last contact’ in several cases, apparently influenced by syntactic cues in that window (e.g., “last time I saw her at work…”) without understanding its actual meaning. Later in the interview (outside the window), the respondent discussed an event more proximal to the suicide.

## Discussion

This study investigated if a large language model can achieve sufficient agreement with qualitative researchers to be integrated with qualitative research procedures in a psychosocial autopsy study. The model performed well in the binary classification task (accuracy: 0.84), proving itself capable of recognizing predefined codes in shorter segments. There was substantial agreement with a researcher (Cohens Kappa: 0.68). Performance was lower with the sliding window technique (accuracy: 0.67). We noted that most errors were false positives in the binary classification and the sliding window tasks. This might be explained by research that suggests LLMs can hallucinate data to conform with a task (16), which results in false positives rather than false negatives. Interestingly, a recent publication instead warns of the propensity of LLMs to generate false negatives more often than false positives (32). We believe that, in a collaborative approach, false positives are preferable to false negatives, because it is less time-consuming to confirm findings from the model compared to identifying what the model missed. An alternative and more optimistic perspective is that the LLM may have labeled data independently, while researchers implicitly weighed the relative compatibility of different codes. For example, if a text segment referred to the preparation for the suicide, researchers often exclusively used a code describing preparation. Meanwhile, the LLM selected any codes relating to behavior and suicide preparation.

The discrepancy in performance between binary classification and data labeling through a sliding window approach stood out. In the latter, we dichotomized the occurrence of a code in a transcript, which obviously favors codes that are salient in the corpus. Codes scoring well in both the binary classification and the sliding window task were characterized by (1) a specific definition, (2) semantic exclusivity (as little overlap with other codes as possible) and (3) moderate occurrence throughout the corpus. An LLM particularly struggles with “other” categories based on negation and does not adequately classify this type of data. Codes and coherent definitions are critical for performance. Codes purposed to identify a specific topic should be split into a list of subcodes, preferably with mutually exclusive definitions to improve accuracy, and they should not be formulated with a negation structure. Although accuracy was lower for the broader codes in binary classification due to the large number of false positives, the summaries were exhaustive. Researchers could consider a stepped summarization procedure to further condense LLM output, but this requires additional scrutiny.

On an hourly scale, the model was not optimized for performance. The model took approximately 5 h per interview (maximum *n* = 30,000 words), which was 1 h longer than a researcher typically needed to code the average interview. One avenue to achieve large reductions in computing time is to use sequential segments instead of a sliding window approach. Properly segmenting an interview beforehand result in roughly four times fewer operations, for segments equal to the window size. This would mean the model could code an interview in slightly over an hour. Another possible time increase could be gained with improvements to the capability of the LLM and its context window, allowing for interviews to be coded in their entirety without segmentation. Moreover, we believe that it is in the broader time schedule of a qualitative research project that Large Language Models will make the difference. The researchers in our project took roughly 4 months to code 38 interviews, as they obviously did not work continuously and needed to balance other work, while the model completed the deductive coding task of all 38 interviews in 8 days.

Qualitative research entails more than coding the data alone. A comparison of the steps after coding—reviewing, interpreting, and processing the findings for a report— was outside the scope of this study. The time investment of these intellectual tasks is conceivably affected by familiarity with the data (which manual coding might foster), how the output is structured, the quality of the output, and many other factors. We believe it would require more rigorous research to properly compare the efficiency of an LLM research assistant to a human researcher, in a design that includes accurate measures of all subprocesses of qualitative analysis but also appreciates the broader time schedule of a study (33).

The contents summarized by the LLM were mostly rated as ‘good’ by researchers. The model provided summaries suitable for subsequent qualitative analysis by a researcher, referring to the original data. The reference to the original data increases transparency, as it indicates how a model came to its output despite operating as a black box and allowing researchers to double check any conclusions made by the LLM. Approximately one fifth of the summaries were rated as ‘poor.’ There was a large discrepancy in quality between different codes. The LLM sometimes summarized inaccurately, using data unrelated to the code definition. These findings align with recent research by Zhang, Jijo and colleagues (34), and are conceivably affected by text structure. Interviewers can additionally be instructed to more strictly adhere to an interview protocol, if required, to improve the interview structure, and ensure that all topics from the instrument are touched. An infrequent but serious error was hallucination. This may have been caused by incomplete or noisy data, unspecific definitions, or semantic gaps. In our qualitative assessment, we noticed that the content of hallucinated data often mirrored syntactic structures in the few-shot examples in our prompts. Future research may determine if prompts combined with few-shot examples can induce hallucinations. High-quality data, specific prompts and techniques such as ‘self-familiarity’ (35) may prevent hallucination to some extent. Finally, the summaries appeared consistent across demographically diverse cases. Our model was not provided with demographic information about the decedent. A different methodology could be more appropriate to rigorously investigate bias of language models in coding tasks. Researchers could, for example, prompt the model with a duplicate interview segment, but provide a different -fabricated- demographic background with each segment to compare and assess the model outputs for structural bias.

## Strengths and limitations

The current study has been among the first to investigate the potential of an LLM to deductively code and summarize complex qualitative data. Performance was high for a first attempt without training and these findings are promising in a reasonably young field of research. There are several limitations of our research. Firstly, the corpus was not large enough to create a dataset with enough training and test examples. A trained model may improve outcomes. Secondly, although state-of-the-art LLMs have increasingly large context lengths [8,192 tokens for LLAMA3 (27)], this is not yet enough to capture an interview of up to 30,000 words. Therefore, our model used a sliding window. This obstructed chronological annotation. For example, capturing the nuances of (changes in) behavior in chronological order in the period preceding suicide was hampered by the windows’ brevity. Chronological classification may become possible as the context size of LLMs increases, and feasible with improved computing power. Lastly, the dataset used in this research was Dutch. Our findings may not directly translate to other languages.

## Conclusion

State-of-the-art language models can support researchers in deductively coding complex interview data. We found some evidence that it improved efficiency, but more research is needed to investigate if large language models can systematically alleviate the investment of time and resources in qualitative research. Additionally, these models may facilitate unprecedented, near real-time monitoring based on qualitative data which can improve the timeliness of public health prevention efforts. Qualitative researchers can thus reinvent their workflow by integrating language models, but they should explicitly retain responsibility for the quality of the output. Based on our findings, we recommend a collaborative approach to strike a balance between efficiency and oversight, whereby the LLM performs initial deductive coding using key indicators and the researchers review the output, interpret the data, and perform additional inductive coding. Ideally, an active learning loop is incorporated into the design, through which researchers can provide feedback to the model about appropriately and inappropriately coded segments. Future research should aim to include a variety of qualitative data, concerning both data structure and subjects, to investigate functionality in diverse contexts.

## Data Availability

The datasets presented in this article are not readily available because interview data used to evaluate the model cannot be shared publicly because of ethical restrictions: the dataset contains potentially identifying and sensitive information, and the Medical Research Ethics Committee (MREC) of Amsterdam UMC has imposed this restriction (registration number: NL76295.029.21). Contact: metc@vumc.nl, https://metc.amsterdamumc.org/. Data may be made available upon reasonable request if the researcher adheres to the criteria formulated by the MREC. Contact the corresponding author (EB) for inquiries. Requests to access the datasets should be directed to e.balt@113.nl.

## Supplementary material

Supplementary File 1 Codes and definitions.

Supplementary File 2 LLM summarization scores illustration.

Supplementary File 3 Server specifications.
