# Supplementary material for: Deductively coding psychosocial autopsy interview data using a few-shot learning large language model
Source: Front Public Health. 2025 Feb 19;13:1512537. doi: 10.3389/fpubh.2025.1512537 (PMC11879832; doi:10.3389/fpubh.2025.1512537)
Supplement: Supplementary File 3 — Server specifications. [file Supplementary_file_3.docx]

**Implementation specifications**

We ran our analysis on a single NVIDIA Tesla A100 40GB GPU. We used Meta's Llama 3 70B instruct version, with 4bit quantization. Inference was done with a temperature of 0.6. The Huggingface Transformer library was used to implement the model.
